# The Listening Zone of Human Electrocorticographic Field Potential Recordings

**DOI:** 10.1523/ENEURO.0492-21.2022

**Published:** 2022-04-21

**Authors:** Meredith J. McCarty, Oscar Woolnough, John C. Mosher, John Seymour, Nitin Tandon

**Affiliations:** 1 Vivian L. Smith Department of Neurosurgery, McGovern Medical School, University of Texas Health Houston, Houston, TX 77030; 2 Texas Institute for Restorative Neurotechnologies, University of Texas Health Science Center at Houston, Houston, TX 77030; 3 Memorial Hermann Hospital, Texas Medical Center, Houston, TX 77030

**Keywords:** electrocorticography, human, intracranial recording, inverse modeling, referencing

## Abstract

Intracranial electroencephalographic (icEEG) recordings provide invaluable insights into neural dynamics in humans because of their unmatched spatiotemporal resolution. Yet, such recordings reflect the combined activity of multiple underlying generators, confounding the ability to resolve spatially distinct neural sources. To empirically quantify the listening zone of icEEG recordings, we computed correlations between signals as a function of distance (full width at half maximum; FWHM) between 8752 recording sites in 71 patients (33 female) implanted with either subdural electrodes (SDEs), stereo-encephalography electrodes (sEEG), or high-density sEEG electrodes. As expected, for both SDEs and sEEGs, higher frequency signals exhibited a sharper fall off relative to lower frequency signals. For broadband high γ (BHG) activity, the mean FWHM of SDEs (6.6 ± 2.5 mm) and sEEGs in gray matter (7.14 ± 1.7 mm) was not significantly different; however, FWHM for low frequencies recorded by sEEGs was 2.45 mm smaller than SDEs. White matter sEEGs showed much lower power for frequencies 17–200 Hz (q < 0.01) and a much broader decay (11.3 ± 3.2 mm) than gray matter electrodes (7.14 ± 1.7 mm). The use of a bipolar referencing scheme significantly lowered FWHM for sEEGs, relative to a white matter reference or a common average reference (CAR). These results outline the influence of array design, spectral bands, and referencing schema on local field potential recordings and source localization in icEEG recordings in humans. The metrics we derive have immediate relevance to the analysis and interpretation of both cognitive and epileptic data.

## Significance Statement

Human intracranial recordings have promising implications for clinical application, neuroscientific research, and the design of brain computer interfaces. However, there are numerous factors that ambiguate the interpretation of these neural recordings in humans, including electrode design and data analysis techniques. The present findings compare the effects of electrode design, frequency of recorded neural activity, and referencing scheme on the listening zone of intracranial recordings. These results suggest referencing scheme and electrode design and location to be critical considerations when analyzing high-frequency recordings. This comprehensive comparison of the listening zone of intracranial recordings is a pivotal step for both future array design and the standardization of current interpretation of human neural activity.

## Introduction

Invasive neural recordings provide a unique window into human cognition. Over the last several decades, intracranial field potential recordings have yielded profound insights into a variety of neural systems (Crone et al., 2006), including speech production (Pasley et al., 2012; Cogan et al., 2014), auditory processing (Sinai et al., 2009; Miller et al., 2021), language (Forseth et al., 2018; Conner et al., 2019), visual perception (Martin et al., 2019), motor control (Salari et al., 2019), decision-making (Bartoli et al., 2018), emotion (Guillory and Bujarski, 2014), and memory (Foster et al., 2012; Derner et al., 2018). An array of electrode designs and recording scales are now being implemented and ongoing progress in neuroengineering is yielding rapid advances in electrode design. The gap between what recording scale is technologically possible and that which is optimal for understanding the neurobiology of cognition, epilepsy, or to provide inputs for brain machine interfaces, remains unknown (Marblestone et al., 2013; Pesaran et al., 2018). Answers to these questions, especially the optimal form factor required to resolve spatially distinct sources within the complex electric field landscape of the brain will influence the design of newer recording interfaces (Cybulski et al., 2015).

The uncertainty of reconstructing the spatial and temporal sources based on multi-electrode field potentials, the inverse source problem (Herreras, 2016; Pesaran et al., 2018) is a direct consequence of the imperfect resolution of recording electrodes and the source properties of the electric field landscape. While the complex geometry of single neurons makes the precise modeling of even one neuron’s activity in isolation difficult to model (Nunez and Srinivasan, 2005), the field potential at any recording electrode is an aggregate of quasi-synchronously active dipoles from a multitude of spatially distributed neural sources (Buzsáki et al., 2012; Łęski et al., 2013). Not all neurons contribute to this electric field landscape at any given instant, and different patterns of neural activity may generate similar field potential measures depending on the distance and the density of recording sites. The neural tissue that comprises this electric field landscape is itself heterogenous, with conductivity and dielectric constants that vary based on cell packing density and cortical location (Nunez and Srinivasan, 2005; Howell and McIntyre, 2016; Bingham et al., 2020).

At the resolution currently provided by macroelectrodes used for human intracranial electroencephalographic (icEEG) recordings, the measured field potential activity is not a direct measure of the activity of local cell assemblies, but rather a larger-scale measure of activity conducted through neural space. This volume conduction can lead to linear relationships between simultaneously recorded signals at neighboring electrodes, and it is hard to disentangle whether high levels of correlated activity between two electrodes are because of underlying neural dynamics (such as common input to or coordination of activity between multiple brain regions; Roelfsema et al., 1997; Womelsdorf et al., 2007; Siegel et al., 2008; Seeber and Michel, 2021) or because of volume conduction of voltage from neighboring regions (Kellis et al., 2016). To resolve this, we define and quantify volume conduction as the instantaneous signal correlation at zero-time lag between electrode pairs, which quantifies common activity because of volume conduction. The lower the instantaneous correlation between electrodes, the lower the signal redundancy of each electrode’s listening zone and the greater its uniqueness. Determining the optimal spacing and location of electrodes to not only minimize signal redundancy, but to also capture separable field potential recordings is a pivotal hurdle for understanding and optimizing invasive field potential recordings in humans (Cybulski et al., 2015).

To investigate the ability of multiple clinically used electrode types in resolving spatially distinct activity, we compared task-related cross-correlations in activity across subdural electrodes (SDEs), stereo-EEG (sEEG) electrodes, and high-density sEEG (hdsEEG) electrodes in patients undergoing monitoring for the localization of medically intractable epilepsy. We analyzed the impact that referencing strategy, electrode location, and frequency components of the signal have on signal redundancy and the influence this could have on neural array design.

## Materials and Methods

### Participants

A total of 71 patients (33 female, 18–65 years) participated in this research after providing written informed consent. All participants were semi-chronically implanted with intracranial electrodes for the localization of pharmaco-resistant epilepsy. All experimental procedures were reviewed and approved by the Committee for the Protection of Human Subjects (CPHS) of the University of Texas Health Science Center at Houston as Protocol Number HSC-MS-06–0385.

### Electrode implantation and data recording

**Figure 1.**
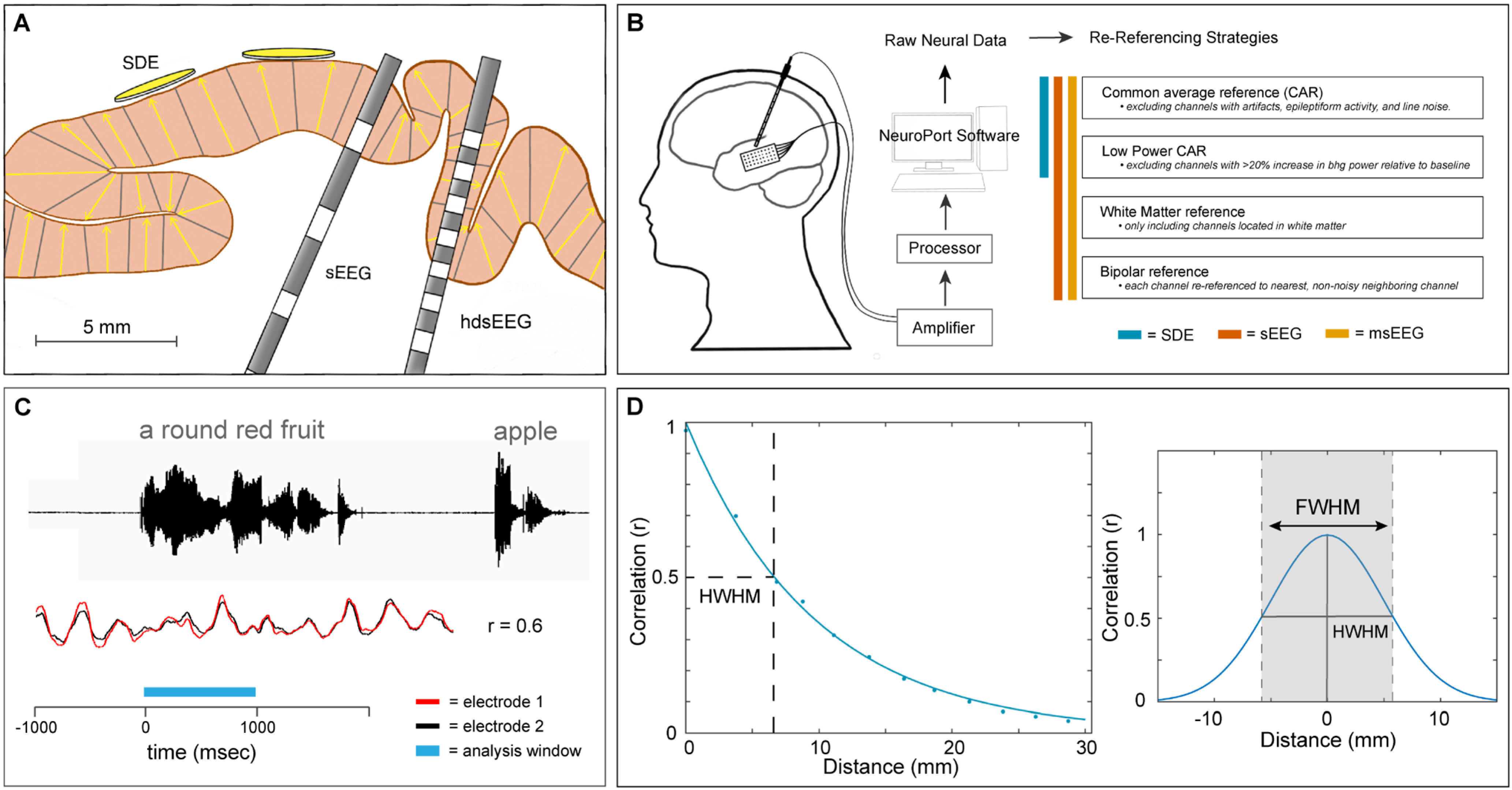
Experimental design. ***A***, Schematic representation of the three electrode scales analyzed: SDEs 3 mm diameter disk, sEEG electrodes 2 mm-long ring, and hdsEEG electrodes 0.5 mm-long ring. sEEG and hdsEEG contacts are depicted in gray. Yellow arrows depict dipole orientation within pictured cortical gray matter. ***B***, Schematic representation of the neural data acquisition and re-referencing strategies. ***C***, Schematic representation of the auditory naming to definition task. Colored bar indicates task-related analysis window (blue; 0–1000 ms), during which cross-correlation (*r*) is calculated between the waveforms of two exemplar neighboring electrodes (red and black; exemplar traces). ***D***, Example of full width at half maximum (FWHM) calculation. The correlation coefficient was measured using the raw voltage of every combination of electrode pairs within 30 mm of each other, for each frequency range. Correlation values were fit with an exponential decay function. Half width at half maximum (HWHM) correlation was measured from this exponential decay function and doubled to generate the FWHM value for each condition.

Data were acquired from either subdural grid electrodes (SDEs; 18 patients), stereotactically placed depth electrodes (sEEGs; 47 patients) or high-density depth electrodes (hdsEEGs; six patients; Fig. 1*A*). SDEs were subdural platinum-iridium electrodes embedded in a silicone elastomer sheet [PMT Corporation; top-hat design; 4.5 mm diameter; 3 mm diameter cortical contact and are embedded in SILASTIC sheets (10 mm center-to-center spacing)], surgically implanted via a craniotomy (Conner et al., 2011; Tandon, 2012; Pieters et al., 2013; Tong et al., 2020). sEEGs were implanted using a Robotic Surgical Assistant (ROSA; Medtech; Tandon et al., 2019; Rollo et al., 2020). Each sEEG probe (PMT corporation, Chanhassen, Minnesota) was 0.8 mm in diameter and had 8–16 electrode contacts. For the standard sEEG electrodes, each contact was a platinum-iridium cylinder, 2.0 mm in length and separated from the adjacent contact by 1.5–2.43 mm. Each patient had 12–20 sEEG probes implanted. For hdsEEG electrodes, each contact was a platinum-iridium cylinder, 0.5 mm in length and separated from the adjacent contact by 0.5 mm. Each hdsEEG probe contained 12 hdsEEG contacts and four sEEG contacts. Each patient had one to four hdsEEG probes implanted. Following surgical implantation, electrodes were localized by co-registration of preoperative anatomic 3T MRI and postoperative CT scans in AFNI (Cox, 1996). Electrode positions were projected onto a cortical surface model generated in FreeSurfer (Dale et al., 1999), and displayed on the cortical surface model for visualization (Pieters et al., 2013). Intracranial data were collected during research experiments starting on the first day after electrode implantation for sEEGs and 2 d after implantation for SDEs. Data were digitized at 2 kHz using the NeuroPort recording system (Blackrock Microsystems), imported into MATLAB, initially referenced to the white matter electrode used as a reference for the clinical acquisition system and visually inspected for line noise, artifacts and epileptic activity. Electrodes with excessive line noise or localized to sites of seizure onset were excluded. Trials contaminated by interictal epileptic spikes were discarded.

### Signal analysis

Across all 75 patients, a total of 2546 SDE, 8493 sEEG, and 204 hdsEEG electrode contacts were implanted. Of these, 704 SDE, 1736 sEEG, and 51 hdsEEG were excluded because of proximity to the seizure onset zone, frequent interictal epileptiform spikes or line noise. The remaining electrodes included were: 1842 SDE, 6757 sEEG, and 153 hdsEEG electrodes. Analyses were performed by bandpass filtering raw icEEG data from each electrode into five frequency bands [θ, 4–8 Hz; α, 8–15 Hz; β, 15–30 Hz; narrowband γ, 30–60 Hz; broadband high γ (BHG), 70–150 Hz]. Line noise was removed using zero-phase second order Butterworth band-stop filters to filter out 60 Hz, as well as 120- and 180-Hz harmonics. Following line noise removal, band-limited voltage traces were obtained (zero-phase third order Butterworth bandpass filters).

### Referencing and re-referencing strategy

During the recording session, a non-noisy clinical hardware reference electrode located in white matter was used as the reference electrode. For analysis, recordings were re-referenced using one of the following schemes (Fig. 1*B*):

#### Common average reference (CAR)

Offline, raw data were visually inspected and electrodes exhibiting electrical noise or epileptiform artifacts were excluded from the common average. Neural data were then re-referenced to the average of all remaining electrodes that were included in this CAR.

#### Low-power CAR

BHG activity (70–150 Hz) was extracted for each time series (using the original clinical reference) using a frequency domain Hilbert transform and the percentage change in power was measured relative to a baseline time window of −500 to −100 ms before stimulus onset. If the percentage change in mean power was <20%, electrodes were included in the low-power CAR signal averaging.

#### White matter referencing

We identified all sEEG and hdsEEG electrodes located in white matter, gray matter, and CSF based on their position relative to their FreeSurfer surfaces and included all white matter located electrodes.

#### Bipolar referencing

For the bipolar re-referencing, each electrode on the sEEG and hdsEEG probes was re-referenced to its closest neighboring non-noisy electrode located on the same probe. Electrodes on the end of the probe or whose nearest neighboring electrode was noisy were excluded from the analysis.

### Experimental design and statistical analyses

#### Experimental task

All patients participated in an auditory naming-to-definition task (Fig. 1*C*; Forseth et al., 2018), producing single word responses to an auditory presented definition. A total of 70+ auditory stimuli (mean 87) were presented to each patient using stereo speakers (44.1 kHz, 15” MacBook Pro 2015; Forseth et al., 2018). Stimuli had an average duration of 1970 ± 360 ms, and an interstimulus interval (ISI) of 5000 ms. The time period of interest for this analysis was from 0 to 1000 ms following auditory stimulus onset.

##### Full width at half-maximum (FWHM) measure

To compare correlation between electrode pairs over distance, we calculated the FWHM correlation. We first identified all non-noisy pairs of electrodes that were <30 mm from each other (in Euclidean distance). Pairwise Pearson’s correlation was calculated between the band-limited voltage traces for all electrode pairs for each trial (Fig. 1*C*). This correlation value was then averaged across all trials to return one correlation value for each electrode pair and frequency range. A decay function was fit to the absolute values of the correlations within each individual patient (Fig. 1*D*). The decay function was defined as *r* = *(1 – β)^d^*, where the correlation *r* decayed based on the decay factor *β* and the distance *d*. The decay factor was optimized using a least-squares fit. From this decay function, we extracted the distance at which the correlation equaled 0.5, half the theoretical maximum correlation (half width at half maximum; HWHM). The HWHM value was doubled to generate the FWHM value for each condition (Fig. 1*D*). For visualization purposes, the absolute values of these correlations for each patient were binned based on Euclidean distance into 2.5 mm bins.

#### Validation on simulated data

Simulated timeseries data were created using the neural digital signal processing toolbox (Cole et al., 2019). A total of 100 unique power law timeseries were generated in each of the five previously described frequency bands of interest with a power-law exponent of −2, a sampling frequency of 2000 Hz, and a simulation time of 1.5 s to account for the removal of filtering edge effects. The Pearson’s correlation coefficient was calculated between each pair of simulated signals to generate the actual correlation measurement. To calculate the reconstructed correlation dataset, timeseries from each frequency range were first combined to generate a summed electric field signal. Analyses were performed by bandpass filtering combined simulated data into the five previous described frequency bands using identical methods to the main analysis. Signals were randomly re-paired to create 75 simulated trials, approximately matching experimental conditions.

#### Power spectral density (PSD) analysis

Thomson’s Slepian multitaper PSD estimate of the signal was calculated. Significant differences between power in gray and white matter was calculated with Wilcoxon sign-rank tests, corrected for multiple comparisons using a Benjamini–Hochberg false detection rate (FDR) threshold of q < 0.01.

#### Linear mixed effects (LME) modelling

LME models were used to incorporate random and fixed-effects into a linear model. Fixed effects in our model were electrode type and frequency band. The random effect in our model was the participant. Electrode type was SDE, sEEG, or hdsEEG. Data were assumed to be normal in distribution for statistical comparison.

#### Data visualization using raincloud plots

Raincloud plots, incorporating raw data points, probability density, and median, mean, confidence intervals, were used to visualize data (Allen et al., 2019). Reported values for each category are median ± interquartile range.

### Code accessibility

The raw datasets generated from this research study are not publicly available because they contain information that is not compliant with HIPAA. Additionally, the human participants from whom the data were collected have not consented to their public release. We have released anonymized summary statistics at https://osf.io/3efdq/.

## Results

We used a correlation-based analysis to compute the falloff of cross-correlation as a function of distance, between pairs of all non-noisy electrodes regardless of cortical location. We constrained our analysis to task-related neural data, based on prior evidence that the spatial spread of correlated activity is lower during activity as opposed to rest (Muller et al., 2016). Importantly, our analyses compare differences in FWHM across referencing conditions, thereby preserving interelectrode distance as a variable. By preserving interelectrode distance in our FWHM measures, we effectively compute a local reduction in correlation, rather than a global reduction, as is captured in other distance-averaged correlation comparisons.

### Effect of electrode scale and signal frequency on listening zone

**Figure 2.**
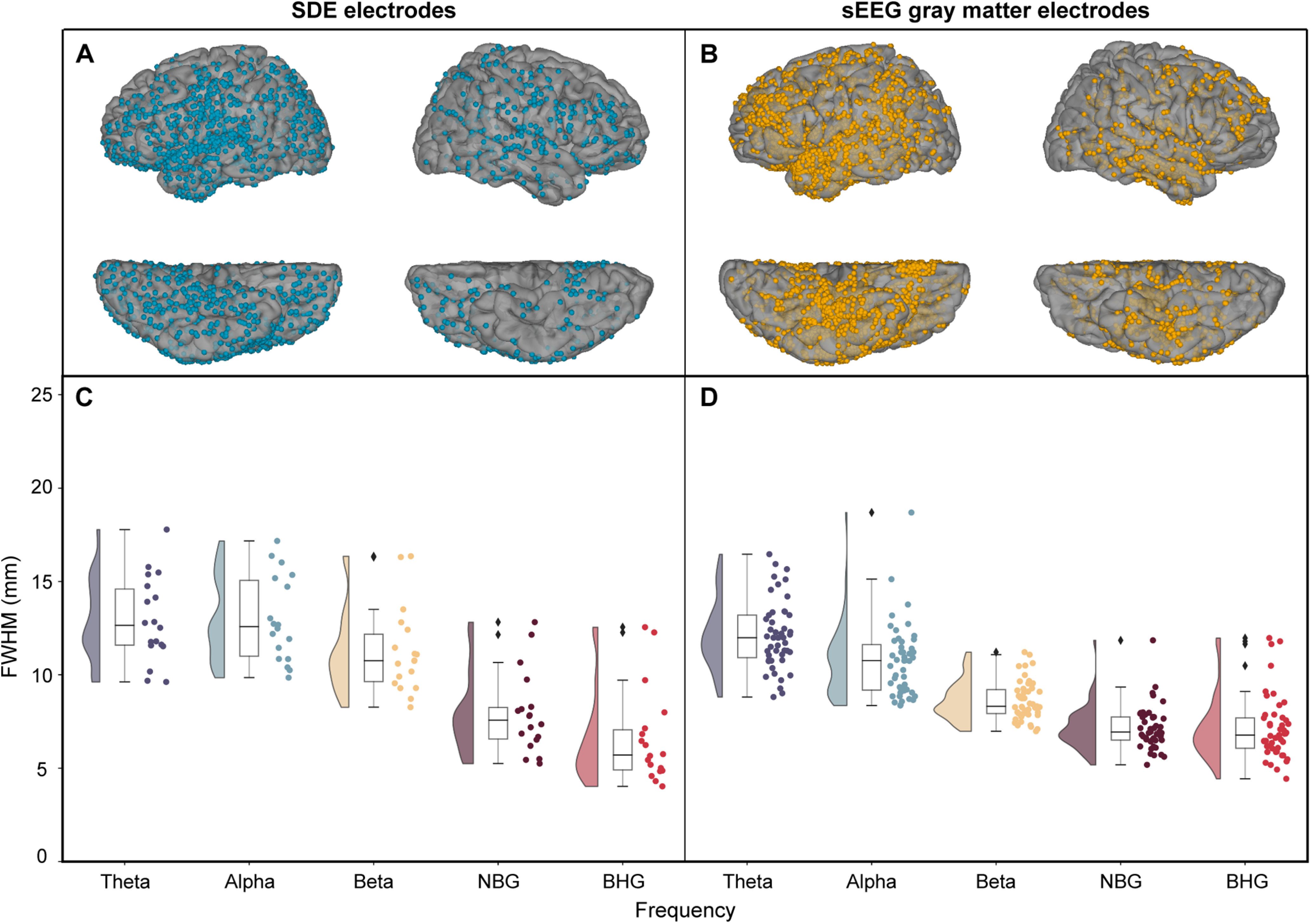
Across-electrode differences in correlation over distance. Coverage map of locations of SDE electrodes (***A***; 18 patients, 1842 electrodes, 37,272 electrode pairs) and gray matter located sEEG electrodes (***B***; 47 patients, 2916 electrodes, 47,522 electrode pairs). Average FWHM was calculated and plotted for each patient for SDE (***C***) and sEEG (***D***) electrode pairs. Frequency ranges of interest: θ (4–8 Hz), α (8–13 Hz), β (13–30 Hz), NBG (30–60 Hz), BHG (70–150 Hz). NBG, narrowband γ; BHG, broadband high γ.

We first compared the decay function (indexed by the FWHM) for SDE versus sEEG electrodes in gray matter, to determine whether a subdural or intracortical location of the icEEG electrode significantly influences the listening zone (Fig. 2). To compare differences in FWHM across frequency and electrode scale, we used a LME model with fixed effects modeling frequency bands and electrode scale (SDE or sEEG). This model explained a large proportion of the variance of FWHM measures (*r*^2^ = 0.65). The electrode type had a significant effect on FWHM (*t*_(321)_ = −4.5, β = −2.4, *p* < 0.001, 95% CI −3.5 to −1.4), which was 2.45 mm smaller for sEEG electrodes than for SDE electrode pairs, when comparing across all frequency ranges. The FWHM of the decay was smaller as frequency increased (LME: *t*_(321)_ = −16.0 β = −1.8, *p* < 0.001, 95% CI −2.0 to −1.6) and there was a significant interaction between frequency and electrode type (*t*_(321)_ = 3.2, β = −0.42, *p* = 0.001, 95% CI 0.17–0.68) indicating that the spatial extent of correlation is significantly dependent on frequency and electrode scale.

For BHG alone, electrode type did not have a significant effect on FWHM (*t*_(34)_ = 1.42, β = 1.07, *p* = 0.17, 95% CI −0.5–2.6). The mean FWHM in BHG for SDE electrodes (6.6 ± 2.5 mm) was slightly lower than for gray matter located sEEG electrodes (7.14 ± 1.7 mm); however, this difference was not significant.

### Location dependence of sEEG electrode listening zone

**Figure 3.**
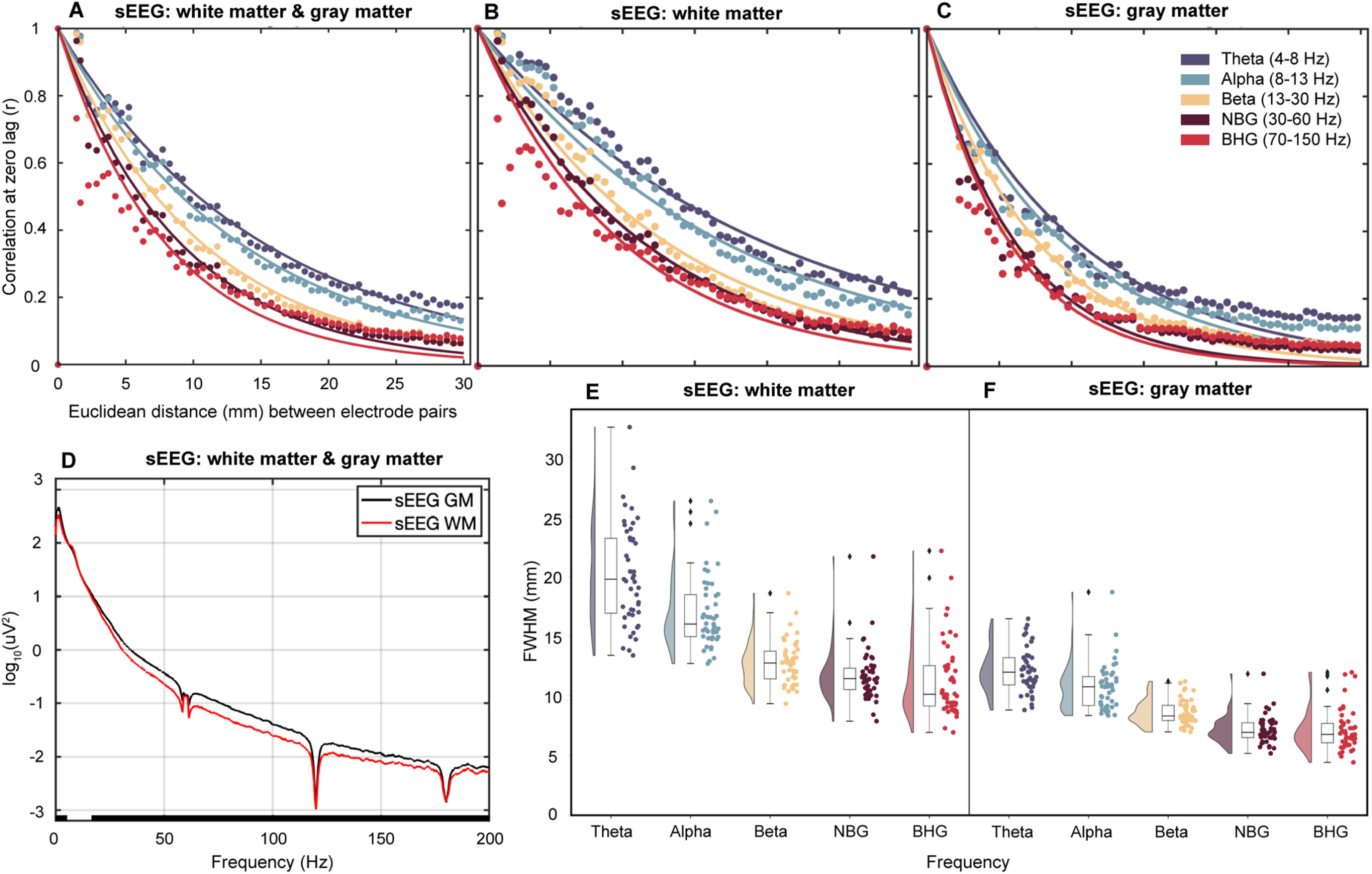
Anatomical location of sEEG contacts in gray or white matter significantly influences FWHM correlation measures. Pearson’s correlation coefficient was measured between pairs of sEEG electrodes located in gray and white matter (***A***; 47 patients; 6757 electrodes; 244,621 electrode pairs), white matter only (***B***; 2649 electrodes; 43,957 electrode pairs), or gray matter only (***C***; 2916 electrodes; 47,522 electrode pairs). Each data point is binned into 0.5 mm bins based on distance between electrode pairs, colored based on frequency range of interest and fit with an exponential decay function shown as colored solid lines. ***D***, Mean PSD plots for sEEG electrodes located in white matter (WM; red; 2649 electrodes) or gray matter (GM; black; 2916 electrodes). Notch filters were applied at 60 Hz and harmonics. Results from Wilcoxon sign-rank test with significance threshold of <0.01 denoted by black bar along the *x*-axis. Raincloud plots depicting FWHM values for each patient in each frequency range for all pairs of white matter located (***E***; 2649 electrodes; 43,957 electrode pairs) and gray matter located (***F***; 2916 electrodes; 47,522 electrode pairs) pairs. Frequency ranges of interest: θ (4–8 Hz), α (8–13 Hz), β (13–30 Hz), NBG (30–60 Hz), BHG (70–150 Hz). NBG, narrowband γ; BHG, broadband high γ.

SDEs sit on the cortical surface, proximal to local field generators, whereas many individual sEEG electrodes are located within white matter, distant from the cortical surface and measuring far field potentials. Thus, the physical location of sEEG electrodes could present potentially confounding correlation measures across distance. An LME model with fixed effects modeling frequency and electrode location (white matter or gray matter located sEEGs) explained a large proportion of the variance in FWHM measures (*r*^2^ = 0.78). sEEG electrodes located in gray matter had a much smaller FWHM (8.3 mm lower) compared with those located in white matter (LME: *t*_(466)_ = −17.3, β = −8.3, *p* < 0.001, 95% CI −9.2 to −7.3; Fig. 3). Additionally, the interaction between FWHM and frequency range significantly depended on electrode location (*t*_(466)_ = 6.6, β = 0.95, *p* < 0.001, 95% CI 0.67–1.2) with low frequencies showing a broader listening zone in white matter electrodes. For θ frequencies, the mean FWHM for sEEG electrodes located in white matter was 20.2 ± 4.3 mm, whereas the mean FWHM for gray matter sEEG electrodes was 12.1 ± 1.8 mm (Fig. 3*E*,*F*).

When comparing the effect of electrode location on BHG activity, an LME model with fixed effects modeling electrode location explained a large proportion of the variance of the FWHM measures (*r*^2^ = 0.85). For BHG frequencies, the mean FWHM for sEEG electrodes located in white matter was 11.3 ± 3.2 mm, whereas the mean FWHM for sEEG electrodes located in gray matter was 7.14 ± 1.7 mm. For the BHG band, electrode location did have a significant effect on FWHM of signal correlation decay (*t*_(92)_ = −13.5, β = −4.2, *p* < 0.001, 95% CI −4.8 to −3.6). Of course, there is not much power in white matter recordings and these correlations may be higher given these lower amplitude signals. To assess this, we compared mean PSD plots for sEEG electrodes located in white matter or gray matter, demonstrating the much lower power in white matter sEEG electrodes (for all frequencies 18–200 Hz; q < 0.01; Fig. 3*D*).

### Referencing strategies for SDE and sEEG electrodes

**Figure 4.**
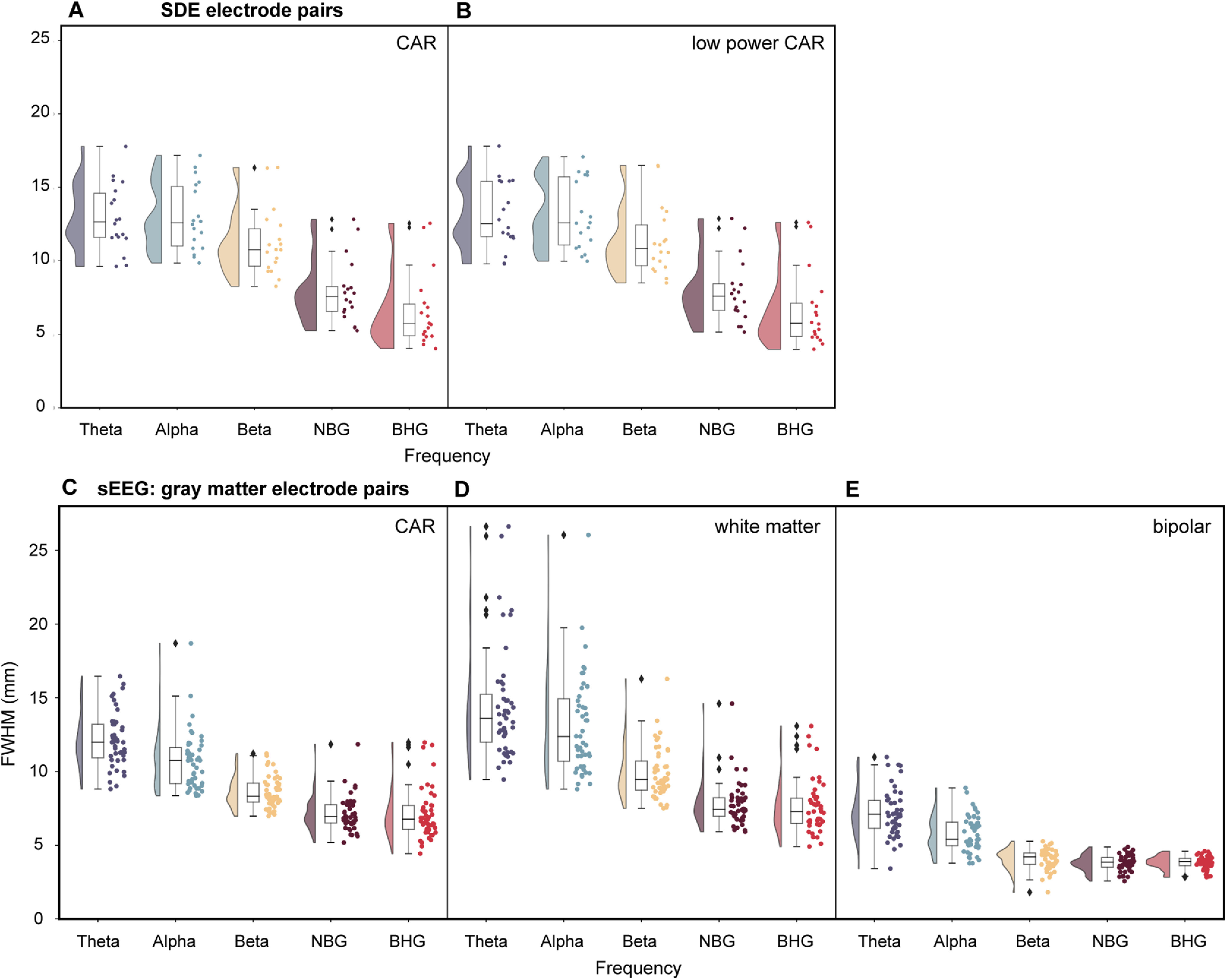
Referencing scheme comparison across SDEs and gray matter located sEEGs. Average FWHM was calculated and plotted for each patient for SDE (***A***, ***B***; *n* = 18 patients) and sEEG (***C–E***; *n* = 47 patients) electrode pairs in each frequency range of interest. For SDE electrode pairs (1842 electrodes; 37,272 electrode pairs), average FWHM was compared using either CAR (***A***) or low-power CAR scheme (***B***). For gray matter located sEEG electrode pairs (2916 electrodes; 47,522 electrode pairs), average FWHM was compared using either CAR (***C***), white matter (***D***), or bipolar referencing schemes (***E***). Frequency ranges of interest: θ (4–8 Hz), α (8–13 Hz), β (13–30 Hz), NBG (30–60 Hz), BHG (70–150 Hz). NBG, narrowband γ; BHG, broadband high γ.

Next, we examined the influence of referencing schemes on measured correlation. Based on evidence that referencing strategies can eliminate or increase spurious correlation between recording electrodes (Li et al., 2018), we compared several commonly used referencing schemes; CAR, low-power CAR, white matter referencing, and bipolar referencing, across SDE and gray matter located sEEG electrode pairs (Fig. 4).

A two-way ANOVA was conducted comparing effects of referencing scheme and frequency range on FWHM measures. For SDE electrode pairs, there was no significant interaction between the referencing scheme and the frequency band on FWHM measures (*F*_(4,170)_ = 0.01, *p* = 0.99). There was a significant effect of frequency (*F*_(4,170)_ = 57.96, *p* < 0.001) on FWHM, but no significant effect of referencing scheme (*F*_(1,170)_ = 0.07, *p* = 0.79). For BHG activity, SDEs showed a correlation decay of 6.6 ± 2.5 mm FWHM for the CAR scheme (Fig. 4*A*), and 6.6 ± 2.6 mm FWHM for the low-power CAR scheme (Fig. 4*B*). For BHG frequency specifically, a two-way ANOVA showed no significant effect of referencing scheme on FWHM for SDE electrodes (*F*_(1,35)_ = 5.5 × 10^−5^, *p* = 0.99). For θ activity, SDEs showed a correlation decay of 13.0 ± 2.3 mm FWHM for the CAR scheme (Fig. 4*A*), and 13.1 ± 2.3 mm FWHM for the low-power CAR scheme (Fig. 4*B*).

For gray matter located sEEG electrode pairs, a two-way ANOVA showed a significant effect of referencing type (*F*_(2,690)_ = 586.48, *p* < 0.001) and frequency (*F*_(4,690)_ = 207.83, *p* < 0.001) on FWHM values. There was a significant interaction between frequency and referencing scheme on FWHM values (*F*_(8,690)_ = 9.92, *p* < 0.001). For BHG activity, sEEGs showed a correlation decay of 7.14 ± 1.7 mm FWHM for the CAR scheme (Fig. 4*C*), 7.62 ± 1.8 mm for the white matter referencing scheme (Fig. 4*D*), and 3.83 ± 0.45 mm for the bipolar referencing scheme (Fig. 4*E*). For BHG frequency specifically, a two-way ANOVA showed a significant effect of referencing scheme on FWHM for sEEG electrodes (*F*_(2,140)_ = 94.4, *p* < 0.001). For θ activity, sEEGs showed a correlation decay of 12.1 ± 1.8 mm FWHM for the CAR scheme (Fig. 4*C*), 14.4 ± 3.8 mm for the white matter referencing scheme (Fig. 4*D*), and 7.19 ± 1.6 mm for the bipolar referencing scheme (Fig. 4*E*).

### Listening zone of hdsEEG electrodes

**Figure 5.**
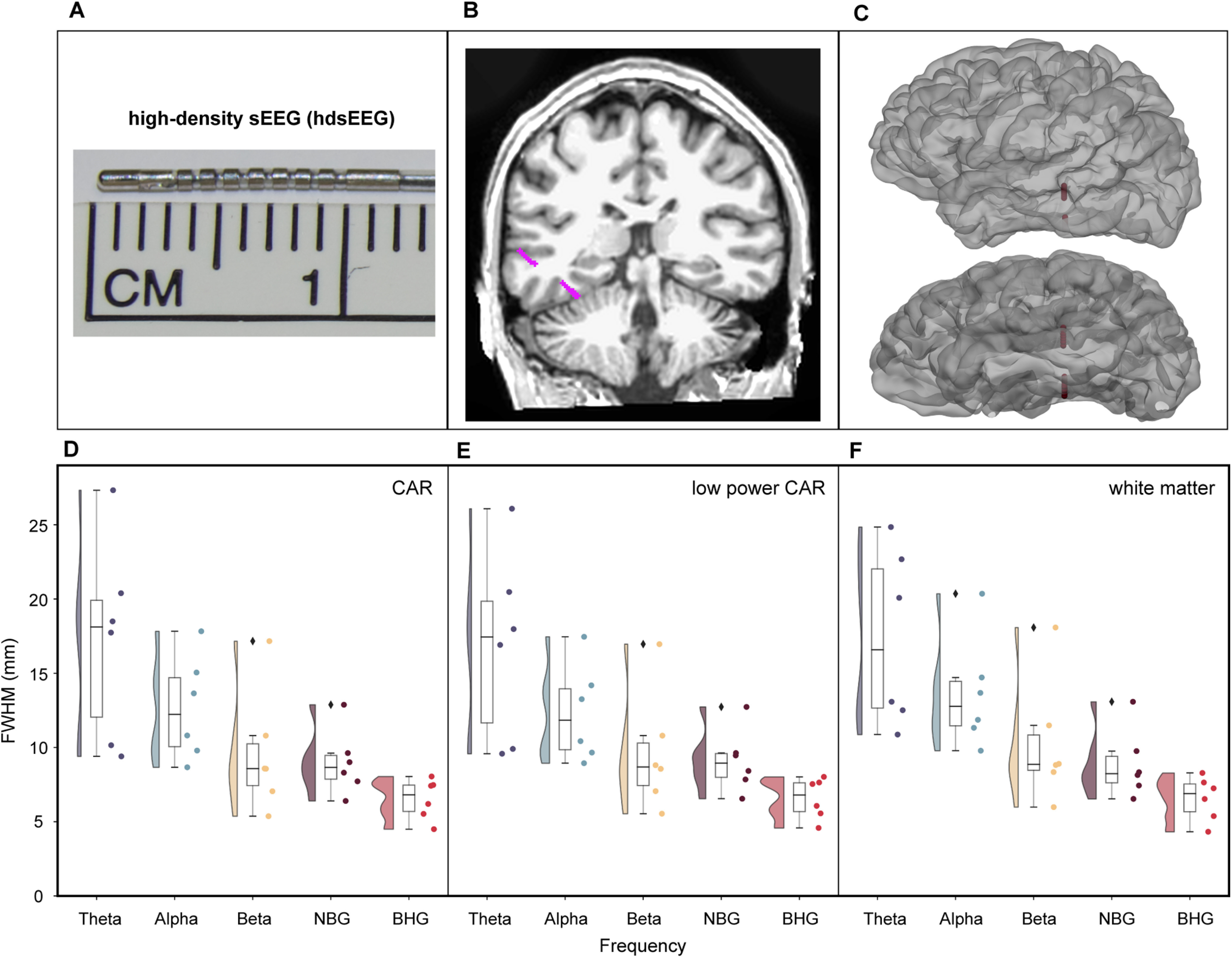
Referencing scheme comparison across hdsEEG electrodes. hdsEEG electrodes are 0.5 mm in length and are on hybrid probes with sEEG 2 mm contacts (***A***). An exemplar hdSEEG electrode is shown in magenta in a coronal MRI slice (***B***) and in a surface reconstruction of the same patient (***C***). Average FWHM was calculated and plotted for each patient for hdsEEG electrode pairs in each frequency range of interest. For hdsEEG electrode pairs (6 patients; 153 electrodes; 1967 electrode pairs), average FWHM was compared using either CAR (***D***), low-power CAR (***E***) or white matter referencing schemes (***F***). Frequency ranges of interest: θ (4–8 Hz), α (8–13 Hz), β (13–30 Hz), NBG (30–60 Hz), BHG (70–150 Hz). NBG, narrowband γ; BHG, broadband high γ.

The final group analysis compared pairwise correlation between hdsEEG electrodes (six patients; 153 electrodes) across referencing scheme. These electrodes were cylinders of 0.5 mm length as compared with 2 mm contacts in standard sEEGs. (Fig. 5*A*). For broadband γ activity, hdsEEG electrode pairs (CAR) had a mean FWHM of 6.5 ± 1.4 mm relative to the FWHM for gray matter located sEEG electrode pairs (7.14 ± 1.7 mm) and the SDE electrode pairs (6.6 ± 2.5 mm). For BHG frequency specifically, a two-way ANOVA showed no significant effect of referencing scheme on FWHM for hdsEEG electrodes (*F*_(2,17)_ = 0, *p* = 0.998). For θ activity, hdsEEG electrode pairs (CAR) had a mean FWHM of 17.3 ± 6.7 mm relative to FWHM for gray matter sEEG electrode pairs (12.1 ± 1.8 mm) and SDE electrode pairs (13.0 ± 2.3 mm).

We compared CAR (Fig. 5*D*), low-power CAR (Fig. 5*E*), and white matter (Fig. 5*F*) referencing schemes for hdsEEG electrode pairs. A two-way ANOVA was conducted comparing effects of referencing scheme and frequency range on FWHM measures. There was no significant interaction between the effects of referencing scheme and frequency range on FWHM measures (*F*_(8,75)_ = 0.04, *p* = 1.0). Higher frequencies had significantly lower FWHM than lower frequencies (*F*_(4,75)_ = 19.59, *p* < 0.001), and referencing scheme had no effect on FWHM measures (*F*_(2,75)_ = 0.11, *p* = 0.90).

### Methodological validation on simulated neural data

**Figure 6.**
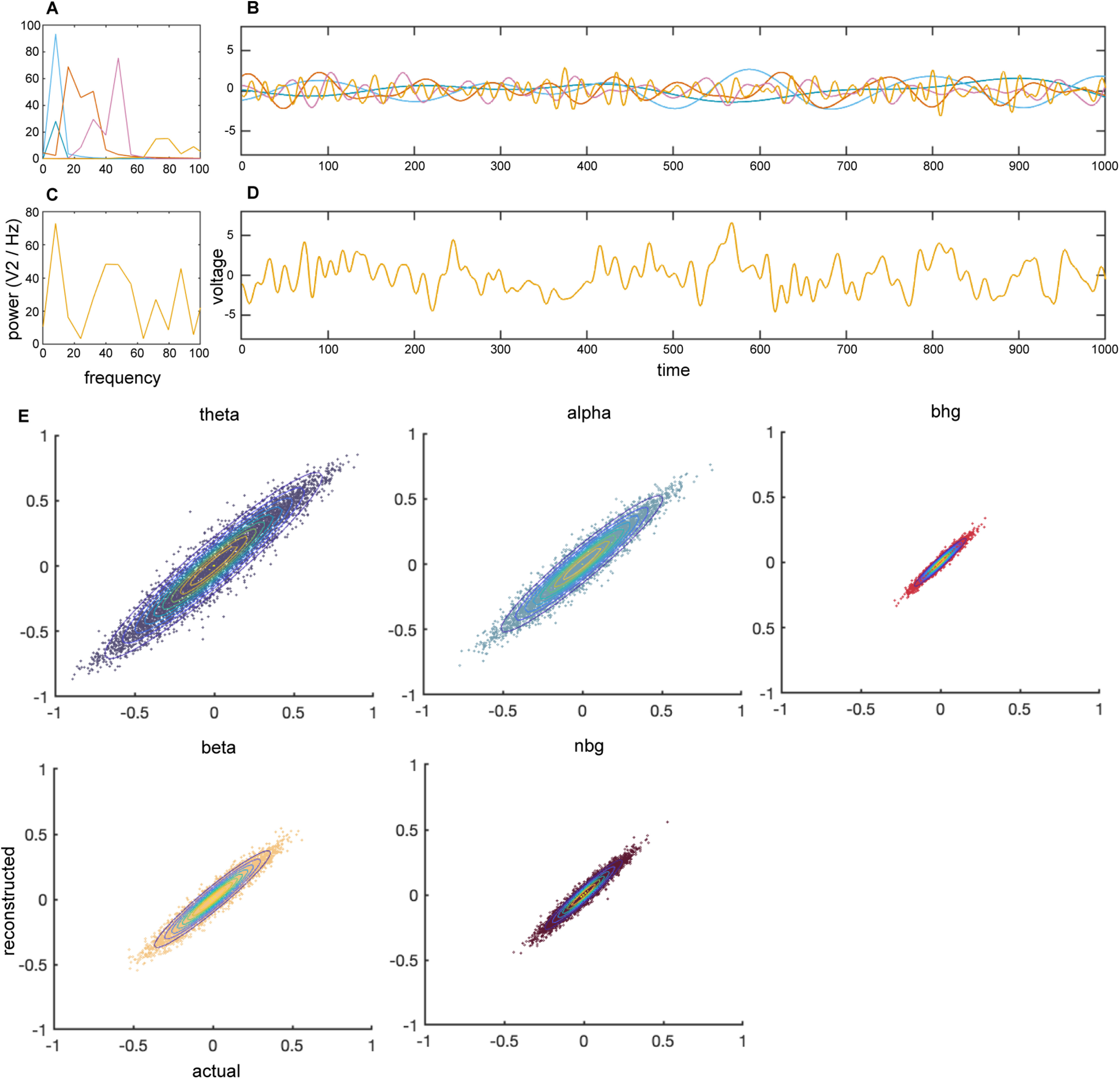
Correlation analysis on simulated timeseries data reveals no spurious correlation because of the analytic pipeline. Representative spectral power (***A***) and timeseries (***B***) of simulated neural data in each frequency range of interest (θ, 4–8 Hz; α, 8–15 Hz; β, 15–30 Hz; narrowband γ, 30–60 Hz; broadband high γ, 70–150 Hz). Representative power spectrum (***C***) and timeseries (***D***) of electric field signal comprised of summed timeseries in each frequency shown in ***B***. Comparison of actual and reconstructed Pearson’s correlation coefficient (*r*) between every combination of simulated timeseries (***E***) overlayed with 2D probability density estimation reveal no significant difference between actual and reconstructed correlation values on simulated data.

To validate our analysis pipeline, we also analyzed simulated neural time series data (Cole et al., 2019) in the known correlation values between narrowband signals (Fig. 6). In comparing the known pairwise correlation (actual) between simulated signals with correlations (reconstructed) after running the simulated signals through our analysis pipeline, we aimed to ensure our analysis pipeline was not itself introducing any unexpected confounds to our neural data analyses. We found no differences between the actual and reconstructed correlations between narrowband signals in each frequency range of interest (Fig. 6*E*).

## Discussion

We have systematically quantified the influence of electrode type, reference scheme and frequency band on the ability to dissociate sources at different scales in human icEEG recordings. Our work shows significant differences in the listening zone across electrode type and frequency band, with SDEs exhibiting the largest listening zone on average relative to sEEGs or hdsEEGs for low frequencies. When considering only high γ activity, the listening zone was comparable across SDE and sEEG electrodes. The location of sEEG electrodes significantly influenced FWHM measures, with sEEG electrodes located in white matter exhibiting lower power and greater FWHM values than those located in gray matter. There is a significant interaction between spectral band and FWHM for all electrode types, with high-frequency γ signals exhibiting faster fall off of correlation over distance relative to lower frequency signals. Referencing schema only had a significant effect on FWHM measures for sEEG electrodes, with bipolar referencing generating significantly lower FWHM measures as compared with common average or white matter referencing.

Our reason for initially deciding to constrain our analysis to task-related neural data were based on previous evidence that the spatial spread of correlated activity is lower during activity as opposed to rest (Muller et al., 2016). We also used the ISI −750 to −250 ms before auditory stimulus onset and compared correlations in this interval with the stimulus period of 0–1000 ms after stimulus onset. This analysis by time period (stimulus vs ISI) on FWHM measures across electrode scale showed that for neither SDE electrodes, sEEG nor hdsEEG electrodes, were significant effects of these time epochs on the FWHM measures of BHG correlation found (SDE: *F*_(1,33)_ = 1.42, *p* = 0.24; sEEG: *F*_(1,92)_ = 0.11, *p* = 0.74; hdsEEG: *F*_(1,10)_ = 0.11, *p* = 0.74). Thus, there are no significant differences in FWHM measures for task induced activation versus the baseline period.

Given that our measures are of an auditory task-related response, the issue of greater coverage by SDE relative to sEEG may be raised. The coverage maps in Figure 2*A*,*B* reveal extensive coverage of auditory and temporal cortex for both SDE and sEEG patient cohorts. Further, previous work using both SDE and sEEG recordings during an auditory task clearly shows that well positioned temporal opercular sEEG electrodes reveal much greater activation than corresponding SDE electrodes, at the group as well as the individual level (Forseth et al., 2020)

### Influence of electrode type on FWHM measures

The location of each electrode, whether atop the cortical surface (SDEs) or intracortically located (sEEGs) led to substantive differences in the listening zone. Across all frequencies, SDEs had broader spread of correlation over distance, with an average FWHM 2.45 mm greater than sEEGs, indicating a more local listening zone for sEEG electrodes. Importantly, the mean FWHM for BHG alone was not significantly different between SDE (6.6 ± 2.5 mm) and sEEGs (7.14 ± 1.7 mm), indicating a preserved locality of BHG across electrode scale (Fig. 2). hdsEEG electrode pairs had mean FWHM of 6.5 ± 1.4 mm for BHG, exhibiting the most local listening zone for correlation over distance, albeit in a smaller patient cohort with less electrode pairs in each patient than the SDE and sEEG comparisons (Fig. 5).

While there is no consensus on the effect that various recording electrodes have on potential distribution, an electrode’s surface impedance, distance from the source, and source strength all affect source localization (Vermaas et al., 2020; Næss et al., 2021; von Ellenrieder et al., 2021). Electrodes act as capacitors, and their size and impedance (the degree of resistance and reactance with surrounding electric potentials) impacts the resolution of the data (Moffit and McIntyre, 2005; Hnazaee et al., 2020). SDE electrodes have 3 mm of cortical contact while the sEEG and hdsEEG electrodes are both 0.8 mm diameter probes with cylinder lengths of 2 versus 0.5 mm, respectively. All three electrode types are made of the same platinum-iridium alloy, which reduces impedance and improve signal-to-noise ratio (Cogan, 2008). Primary considerations for recording electrodes include the impedance because of electrode contact surface (in this case, cylindrical vs top-hat design) and because of electrode material (identical platinum-iridium in this case). The SDE, sEEG, and hdSEEG electrodes examined here all have varying electrode size, orientation, spacing, and cortical location, which introduces distinct physical differences in resolution and listening zone, especially when considering activity in lower frequency ranges.

### Interaction between spectral band and FWHM measures

Across electrode scale, high-frequency γ signals exhibited a faster fall off of correlation values across distance, consistent with a smaller spatial reach of a local, weaker, and less synchronous high-frequency γ signal (Łęski et al., 2013; Dubey and Ray, 2020; von Ellenrieder et al., 2021). This is concordant with synchronous low frequency activity engaging a larger neural substrate than more focal and transient high-frequency activity (Lachaux et al., 2012; Rouse et al., 2016; Parvizi and Kastner, 2018; Torres et al., 2019). Interestingly, this fall off of correlation values at lower frequencies varied across electrode type. The mean FWHM for BHG for gray matter sEEG electrodes (7.14 ± 1.7 mm), SDE electrodes (6.6 ± 2.5 mm), and hdsEEG electrodes (6.5 ± 1.4 mm) were close in value, whereas the mean FWHM for θ for hdsEEG electrodes (17.3 ± 6.7 mm) was greater than FWHM for SDEs (13.0 ± 2.3 mm) and gray matter sEEGs (12.1 ± 1.8 mm; Fig. 2*C*,*D*).

### sEEG electrode location in white or gray matter influences FWHM measures

Signal attenuation is dependent on the conductivity ratio of the medium (Rogers et al., 2020), and white matter is considered largely anisotropic (Nunez and Srinivasan, 2005), especially at this scale of field potential recording (Howell and McIntyre, 2016). As such, white matter has been found to reflect activity from distant gray matter signals as well as volume conduction from nearby gray matter, thus increasing the likelihood of spurious correlation with activity in adjacent or distant regions (Mercier et al., 2017). While average FWHM was significantly greater for white matter located sEEGs (BHG: 11.3 ± 3.2 mm) than gray matter located sEEGs (BHG: 7.14 ± 1.7 mm; Fig. 3*E*,*F*), the average power of activity recorded at white matter located sEEG electrodes was significantly lower than gray matter located electrodes (Fig. 3*D*). This is consistent with previous findings that electrodes located farther from gray matter signal generators record lower amplitude signals (Mercier et al., 2017; Young et al., 2019). As such, the current analyses considered only gray matter located sEEG electrodes to avoid confounds in measures of correlation over distance because of signal attenuation.

### Impact of referencing schema on FWHM measures

Referencing schemes have an often-understated impact on signal detection, and the process of referencing neural signals has been found to distort and artificially inflate neural activation, functional connectivity and other measures (Liu et al., 2015; Mercier et al., 2017; Li et al., 2018). While measures of correlation should be scale-independent, the process of re-referencing likely influences correlation measures because of a decrease in distant noise, aiding in improved signal-to-noise ratio between nearby electrode pairs (Hnazaee et al., 2020). In our data, referencing scheme did not significantly influence FWHM measures for SDE or hdsEEG electrode pairs. However, for sEEG electrode pairs, we found a significant effect of referencing scheme on FWHM measures (Fig. 4*D*,*E*). We found the choice of bipolar referencing scheme generates significantly lower FWHM measures between proximal sEEG electrode pairs, as compared with CAR and white matter referencing. These results corroborate previous findings (Li et al., 2018) comparing the effect of referencing method on Pearson’s correlation values averaged across sEEG electrode pairs regardless of interelectrode distance.

While common average referencing is commonly implemented in icEEG analyses, there are many considerations when implementing a bipolar referencing scheme (Mercier et al., 2017; Li et al., 2018). Bipolar referencing removes all signal common to neighboring electrodes, but this does not consider anatomic location or dipole orientation, which can distort source localization (Hu et al., 2010). Depending on the location and orientation of sEEG electrodes relative to sulci and sources, bipolar referencing could have quite a variable effect on signal detection. Additionally, it is common when analyzing icEEG datasets to combine activity recorded via SDE and sEEG electrodes. In this case, the question of how to implement bipolar referencing in SDE electrodes becomes geometrically complex.

### Comparison with previous studies

From neuroscientific research to the continuing development of brain-computer interfaces, decoding neural activity remains a necessary and complex goal. As neural interfaces continue to develop and our ability to record electrical activity from the brain at smaller scales advances, the overlap between what is feasible and what is informative remains unclear.

There is currently no consensus in how to best allocate activity recorded by various electrode types to regions of nearby cortical space. In the absence of a solution to this problem, current methodologies implemented rely on assumptions and in vivo measurements to model the dielectric, conductive, and anisotropic aspects of neural tissue (Howell and McIntyre, 2016, 2017; Miceli et al., 2017). These include spatial discrimination techniques (Herreras, 2016), surface-based estimates of the recording zone (Kadipasaoglu et al., 2014, 2015) and weighting functions based on electrode properties of size, layout, and impedance (Dubey and Ray, 2019). Computational models incorporating heterogeneity and anisotropy have been found to more accurately reconstruct neural response to stimulation in DBS application (Åström et al., 2012; Howell and McIntyre, 2017).

Unlike work in nonhuman primates (Xing et al., 2009; Dubey and Ray, 2019, 2020), the location and design of neural probes in humans are largely limited to clinical application, making confident parameterization difficult. Despite these limitations, previous research has compared recording scale in humans (Kellis et al., 2016; Muller et al., 2016; Halgren et al., 2018; Lai et al., 2018; Trumpis et al., 2021) to disambiguate the uncertain properties of neural activity captured by different electrodes.

Modern icEEG recordings incorporate data from varying recording scales, cortical locations, referencing strategies, and analysis approaches. There is a wealth of existing data that have been gathered with a variety of tools and methodologies; the question becomes, how can findings be integrated across this diversity of scales? As with all aspects of scientific research, it is only through understanding the limitations of the tools we have to observe neural phenomenon that we can optimize the strengths and get closer to understanding complex aspects of human cognition.

## Synthesis

Reviewing Editor: Christoph Michel, Universite de Geneve Decisions are customarily a result of the Reviewing Editor and the peer reviewers coming together and discussing their recommendations until a consensus is reached. When revisions are invited, a fact-based synthesis statement explaining their decision and outlining what is needed to prepare a revision will be listed below. The following reviewer(s) agreed to reveal their identity: Martin Seeber, Kirill Nourski. Note: If this manuscript was transferred from JNeurosci and a decision was made to accept the manuscript without peer review, a brief statement to this effect will instead be what is listed below.

Both reviewers judged the paper interesting and original and that it helps future researchers using this type of recordings to interpret their results.

However, several points were raised that need some additional analysis and further clarifications.

Major points:

1. Correlations with zero phase lag are taken as result of volume conduction and thus define the spatial spread. However, there is ample evidence that genuine physiological zero-lag correlations exist that might lead to an overestimation of the spatial spread (e.g. Contreras and Steriade, 1996; Roelfsema et al., 1997; Womelsdorf et al., 2007; Siegel et al., 2008; Hipp et al., 2011; Seeber & Michel, 2021). Please discuss the possibility of genuine zero-lag functional coupling affecting the correlation values.

2. The analysis has been performed on signals recorded while participants were involved in an auditory speech processing task. This could have caused a systematic bias in the data because of the differences in electrode coverage between SDE vs sEEG. Specifically, the reviewer wonders what if SDE generally provides a more extensive coverage of regions involved in auditory speech processing (and thus having more robust responses to the stimuli) compared to sEEG (or vice versa). Is it possible to perform the analysis on spontaneous resting-state data?

3. The reasoning and the use of the simulations should be further developed. As it is presented, the simulations were only used to validate the analysis pipeline.

4. For fixed time windows signals with slow frequencies tend to be higher correlated by chance than signals with high frequencies. This is somewhat already visible in the simulated data in Fig.6 where theta and alpha cover larger ranges that higher frequencies.

It is recommended to compute the average of ∼75 correlation values of simulated data, to obtain an estimate of chance correlation (for each f-band) given this time window and number of trials. In that manner it could be assured that the higher correlations for lower frequencies is a physiological effect.

5. Several reference schemes are compared. However, one of the most basic, the reference to a subgaleal or subcutaneous electrode is missing. We recommend to include this reference scheme or at least comment on it?

Minor points:

1. Pg.23, 450: The size of the contacts (which differs across electrode types) might be worth to discuss separately here. In relation to this comment, it would be great to investigate or at least discuss the frequency distribution picked up by the different electrode types.

2. Fig. 1D suggests that some subjects were implanted with a combination of SDE and sEEG arrays? Was it indeed the case, and if so, how many, and did such subjects contribute data to both SDE and sEEG listening zone analysis?

3. The authors define the listening zone in terms of FWHM, which is a metric derived from their analysis approach. I think for general readership, expressing the “listening zone” size in terms of radius or diameter instead would be more intuitive.

4. It would be appropriate to acknowledge earlier work by Nathan Crone and his JHU colleagues on this topic (e.g. Crone NE, Sinai A, Korzeniewska A. High-frequency gamma oscillations and human brain mapping with electrocorticography. Prog Brain Res. 2006;159:275-95. doi:10.1016/S0079-6123(06)59019-3. PMID: 17071238)

5. L102: Panels of Fig 1 are introduced out of order in the text (C, D, B, A, E). Consider either restructuring the Methods section or re-ordering the panels in the figure.

6. L103: Please specify inter-electrode distance in SDE arrays and the type/configuration of arrays used (e.g. 4x1 strips, 4x8 grids, etc).

7. L111: How many contacts per array did hdsEEG electrodes have?

8. L135: Please state that for line noise removal, band-stop filters were applied to filter out 60 Hz as well as 120 and 180 Hz harmonics (per Fig 3D).

9. Fig 1E: Please label axes in the inset.

10. Fig 2C,D (and other similar panels in later figures). It would be helpful to have frequency bands labeled on the X axis (i.e.) underneath the distributions rather than in a legend. Otherwise, it’s not immediately obvious what is the X axis in these figures.

11. Fig 5A: This panel is visually dominated by the ruler with two different scales, and the electrode array itself is not clearly visible. It may work better to have an image of the array and just a scale bar (0.5 cm or 1 cm) next to it for reference.

12. Fig 5B: The caption refers to one exemplar hdsEEG electrode, yet there appear to be two clusters of yellow symbols. Also, it might work better to show the electrode trajectory in a coronal MRI slice rather than a semi-transparent brain. In the present form, it’s not clear how deep the electrode is implanted, which structure does it ultimately target, and all the other arrays are somewhat distracting without adding much to the story.

## References

[B1] Allen M, Poggiali D, Whitaker K, Marshall TR, Kievit RA (2019) Raincloud plots: a multi-platform tool for robust data visualization. Wellcome Open Res 4:63. 10.12688/wellcomeopenres.15191.1 31069261 PMC6480976

[B2] Åström M, Lemaire J-J, Wårdell K (2012) Influence of heterogeneous and anisotropic tissue conductivity on electric field distribution in deep brain stimulation. Med Biol Eng Comput 50:23–32. 10.1007/s11517-011-0842-z 22101515

[B3] Bartoli E, Conner CR, Kadipasaoglu CM, Yellapantula S, Rollo MJ, Carter CS, Tandon N (2018) Temporal dynamics of human frontal and cingulate neural activity during conflict and cognitive control. Cereb Cortex 28:3842–3856. 10.1093/cercor/bhx245 29028974 PMC6188556

[B4] Bingham CS, Paknahad J, Girard CBC, Loizos K, Bouteiller J-MC, Song D, Lazzi G, Berger TW (2020) Admittance method for estimating local field potentials generated in a multi-scale neuron model of the hippocampus. Front Comput Neurosc 14:72. 10.3389/fncom.2020.00072 PMC7417331 32848687

[B5] Buzsáki G, Anastassiou CA, Koch C (2012) The origin of extracellular fields and currents — EEG, ECoG, LFP and spikes. Nat Rev Neurosci 13:407–420. 10.1038/nrn3241 22595786 PMC4907333

[B6] Cogan GB, Thesen T, Carlson C, Doyle W, Devinsky O, Pesaran B (2014) Sensory–motor transformations for speech occur bilaterally. Nature 507:94–98. 10.1038/nature12935 24429520 PMC4000028

[B7] Cogan SF (2008) Neural stimulation and recording electrodes. Annu Rev Biomed Eng 10:275–309. 10.1146/annurev.bioeng.10.061807.160518 18429704

[B8] Cole S, Donoghue T, Gao R, Voytek B (2019) NeuroDSP: a package for neural digital signal processing. J Open Source Softw 4:1272. 10.21105/joss.01272

[B9] Conner CR, Ellmore TM, DiSano MA, Pieters TA, Potter AW, Tandon N (2011) Anatomic and electro-physiologic connectivity of the language system: a combined DTI-CCEP study. Comput Biol Med 41:1100–1109. 10.1016/j.compbiomed.2011.07.008 21851933 PMC3223284

[B10] Conner CR, Kadipasaoglu CM, Shouval HZ, Hickok G, Tandon N (2019) Network dynamics of Broca’s area during word selection. PLoS One 14:e0225756. 10.1371/journal.pone.0225756 31860640 PMC6924671

[B11] Cox RW (1996) AFNI: software for analysis and visualization of functional magnetic resonance neuroimages. Comput Biomed Res 29:162–173. 10.1006/cbmr.1996.0014 8812068

[B12] Crone NE, Sinai A, Korzeniewska A (2006) High-frequency gamma oscillations and human brain mapping with electrocorticography. Prog Brain Res 159:275–295. 10.1016/S0079-6123(06)59019-3 17071238

[B13] Cybulski TR, Glaser JI, Marblestone AH, Zamft BM, Boyden ES, Church GM, Kording KP (2015) Spatial information in large-scale neural recordings. Front Comput Neurosci 8:172. 25653613 10.3389/fncom.2014.00172 PMC4301009

[B14] Dale AM, Fischl B, Sereno MI (1999) Cortical surface-based analysis I. Segmentation and surface reconstruction. Neuroimage 9:179–194. 10.1006/nimg.1998.0395 9931268

[B15] Derner M, Chaieb L, Surges R, Staresina BP, Fell J (2018) Modulation of Item and source memory by auditory beat stimulation: a pilot study with intracranial EEG. Front Hum Neurosci 12:500. 10.3389/fnhum.2018.00500 30618681 PMC6297717

[B16] Dubey A, Ray S (2019) Cortical electrocorticogram (ECoG) is a local signal. J Neurosci 39:4299–4311. 10.1523/JNEUROSCI.2917-18.2019 30914446 PMC6538865

[B17] Dubey A, Ray S (2020) Comparison of tuning properties of gamma and high-gamma power in local field potential (LFP) versus electrocorticogram (ECoG) in visual cortex. Sci Rep 10:5422. 10.1038/s41598-020-61961-9 32214127 PMC7096473

[B18] Forseth KJ, Kadipasaoglu CM, Conner CR, Hickok G, Knight RT, Tandon N (2018) A lexical semantic hub for heteromodal naming in middle fusiform gyrus. Brain 141:2112–2126. 10.1093/brain/awy120 29860298 PMC6365955

[B19] Forseth KJ, Hickok G, Rollo PS, Tandon N (2020) Language prediction mechanisms in human auditory cortex. Nat Commun 11:5240. 10.1038/s41467-020-19010-6 33067457 PMC7567874

[B20] Foster BL, Dastjerdi M, Parvizi J (2012) Neural populations in human posteromedial cortex display opposing responses during memory and numerical processing. Proc Natl Acad Sci U S A 109:15514–15519. 10.1073/pnas.1206580109 22949666 PMC3458396

[B21] Guillory SA, Bujarski KA (2014) Exploring emotions using invasive methods: review of 60 years of human intracranial electrophysiology. Soc Cogn Affect Neurosci 9:1880–1889. 10.1093/scan/nsu002 24509492 PMC4249472

[B22] Halgren M, Fabó D, Ulbert I, Madsen JR, Erőss L, Doyle WK, Devinsky O, Schomer D, Cash SS, Halgren E (2018) Superficial slow rhythms integrate cortical processing in humans. Sci Rep 8:2055. 10.1038/s41598-018-20662-0 29391596 PMC5794750

[B23] Herreras O (2016) Local field potentials: myths and misunderstandings. Front Neural Circuit 10:101. 10.3389/fncir.2016.00101 PMC5156830 28018180

[B24] Hnazaee MF, Wittevrongel B, Khachatryan E, Libert A, Carrette E, Dauwe I, Meurs A, Boon P, Roost DV, Hulle MMV (2020) Localization of deep brain activity with scalp and subdural EEG. Neuroimage 223:117344. 10.1016/j.neuroimage.2020.117344 32898677

[B25] Howell B, McIntyre CC (2016) Analyzing the tradeoff between electrical complexity and accuracy in patient-specific computational models of deep brain stimulation. J Neural Eng 13:e036023. 10.1088/1741-2560/13/3/036023 27172137 PMC5259803

[B26] Howell B, McIntyre CC (2017) Role of soft-tissue heterogeneity in computational models of deep brain stimulation. Brain Stimul 10:46–50. 10.1016/j.brs.2016.09.001 27720186 PMC5241242

[B27] Hu S, Stead M, Dai Q, Worrell GA (2010) On the recording reference contribution to EEG correlation, phase synchrony, and coherence. IEEE Trans Syst Man Cybern B Cybern 40:1294–1304. 10.1109/TSMCB.2009.2037237 20106746 PMC2891575

[B28] Kadipasaoglu CM, Baboyan VG, Conner CR, Chen G, Saad ZS, Tandon N (2014) Surface-based mixed effects multilevel analysis of grouped human electrocorticography. Neuroimage 101:215–224. 10.1016/j.neuroimage.2014.07.006 25019677

[B29] Kadipasaoglu CM, Forseth K, Whaley M, Conner CR, Rollo MJ, Baboyan VG, Tandon N (2015) Development of grouped icEEG for the study of cognitive processing. Front Psychol 6:1008. 26257673 10.3389/fpsyg.2015.01008 PMC4508923

[B30] Kellis S, Sorensen L, Darvas F, Sayres C, O’Neill K, Brown RB, House P, Ojemann J, Greger B (2016) Multi-scale analysis of neural activity in humans: implications for micro-scale electrocorticography. Clin Neurophysiol 127:591–601. 10.1016/j.clinph.2015.06.002 26138146

[B31] Lachaux JP, Axmacher N, Mormann F, Halgren E, Crone NE (2012) High-frequency neural activity and human cognition: past, present and possible future of intracranial EEG research. Prog Neurobiol 98:279–301. 10.1016/j.pneurobio.2012.06.008 22750156 PMC3980670

[B32] Lai M, Demuru M, Hillebrand A, Fraschini M (2018) A comparison between scalp- and source-reconstructed EEG networks. Sci Rep 8:12269. 10.1038/s41598-018-30869-w 30115955 PMC6095906

[B33] Łęski S, Lindén H, Tetzlaff T, Pettersen KH, Einevoll GT (2013) Frequency dependence of signal power and spatial reach of the local field potential. PLoS Comput Biol 9:e1003137. 10.1371/journal.pcbi.1003137 23874180 PMC3715549

[B34] Li G, Jiang S, Paraskevopoulou SE, Wang M, Xu Y, Wu Z, Chen L, Zhang D, Schalk G (2018) Optimal referencing for stereo-electroencephalographic (SEEG) recordings. Neuroimage 183:327–335. 10.1016/j.neuroimage.2018.08.020 30121338 PMC6495648

[B35] Liu Y, Coon WG, de Pesters A, Brunner P, Schalk G (2015) The effects of spatial filtering and artifacts on electrocorticographic signals. J Neural Eng 12:e056008. 10.1088/1741-2560/12/5/056008 26268446 PMC5485665

[B36] Marblestone AH, Zamft BM, Maguire YG, Shapiro MG, Cybulski TR, Glaser JI, Amodei D, Stranges PB, Kalhor R, Dalrymple DA, Seo D, Alon E, Maharbiz MM, Carmena JM, Rabaey JM, Boyden ES, Church GM, Kording KP (2013) Physical principles for scalable neural recording. Front Comput Neurosc 7:137. 10.3389/fncom.2013.00137 PMC3807567 24187539

[B37] Martin AB, Yang X, Saalmann YB, Wang L, Shestyuk A, Lin JJ, Parvizi J, Knight RT, Kastner S (2019) Temporal dynamics and response modulation across the human visual system in a spatial attention task: an ECoG study. J Neurosci 39:333–352. 10.1523/JNEUROSCI.1889-18.2018 30459219 PMC6325255

[B38] Mercier MR, Bickel S, Megevand P, Groppe DM, Schroeder CE, Mehta AD, Lado FA (2017) Evaluation of cortical local field potential diffusion in stereotactic electro-encephalography recordings: a glimpse on white matter signal. Neuroimage 147:219–232. 10.1016/j.neuroimage.2016.08.037 27554533

[B39] Miceli S, Ness TV, Einevoll GT, Schubert D (2017) Impedance spectrum in cortical tissue: implications for propagation of LFP signals on the microscopic level. eNeuro 4:ENEURO.0291-16.2016. 10.1523/ENEURO.0291-16.2016 PMC5282548 28197543

[B40] Miller CA, Behroozmand R, Etler CP, Nourski KV, Reale RA, Oya H, Kawasaki H, Greenlee JDW (2021) Neural correlates of vocal auditory feedback processing: unique insights from electrocorticography recordings in a human cochlear implant user. eNeuro 8:ENEURO.0181-20.2020. 10.1523/ENEURO.0181-20.2020 PMC7877459 33419861

[B41] Moffit MA, McIntyre CC (2005) Model-based analysis of cortical recording with silicon microelectrodes. Clin Neurophysiol 116:2240–2250. 10.1016/j.clinph.2005.05.018 16055377

[B42] Muller L, Hamilton LS, Edwards E, Bouchard KE, Chang EF (2016) Spatial resolution dependence on spectral frequency in human speech cortex electrocorticography. J Neural Eng 13:e056013. 10.1088/1741-2560/13/5/056013 PMC5081035 27578414

[B43] Næss S, Halnes G, Hagen E, Hagler DJ, Dale AM, Einevoll GT, Ness TV (2021) Biophysically detailed forward modeling of the neural origin of EEG and MEG signals. Neuroimage 225:117467. 10.1016/j.neuroimage.2020.117467 33075556

[B44] Nunez PL, Srinivasan R (2005) Electric fields of the brain: the Neurophysics of EEG, Ed 2. Oxford: Oxford University Press.

[B45] Parvizi J, Kastner S (2018) Promises and limitations of human intracranial electroencephalography. Nat Neurosci 21:474–483. 10.1038/s41593-018-0108-2 29507407 PMC6476542

[B46] Pasley BN, David SV, Mesgarani N, Flinker A, Shamma SA, Crone NE, Knight RT, Chang EF (2012) Reconstructing speech from human auditory cortex. PLoS Biol 10:e1001251. 10.1371/journal.pbio.1001251 22303281 PMC3269422

[B47] Pesaran B, Vinck M, Einevoll GT, Sirota A, Fries P, Siegel M, Truccolo W, Schroeder CE, Srinivasan R (2018) Investigating large-scale brain dynamics using field potential recordings: analysis and interpretation. Nat Neurosci 21:903–919. 10.1038/s41593-018-0171-8 29942039 PMC7386068

[B48] Pieters TA, Conner CR, Tandon N (2013) Recursive grid partitioning on a cortical surface model: an optimized technique for the localization of implanted subdural electrodes. J Neurosurg 118:1086–1097. 10.3171/2013.2.JNS121450 23495883

[B49] Roelfsema PR, Engel AK, König P, Singer W (1997) Visuomotor integration is associated with zero time-lag synchronization among cortical areas. Nature 385:157–161. 10.1038/385157a0 8990118

[B50] Rogers N, Thunemann M, Devor A, Gilja V (2020) Impact of brain surface boundary conditions on electrophysiology and implications for electrocorticography. Front Neurosci 14:763. 10.3389/fnins.2020.00763 32903652 PMC7438758

[B51] Rollo PS, Rollo MJ, Zhu P, Woolnough O, Tandon N (2020) Oblique trajectory angles in robotic stereo-electroencephalography. J Neurosurg 135:245–254. 10.3171/2020.5.JNS20975 32796145

[B52] Rouse AG, Williams JJ, Wheeler JJ, Moran DW (2016) Spatial co-adaptation of cortical control columns in a micro-ECoG brain–computer interface. J Neural Eng 13:e056018. 10.1088/1741-2560/13/5/056018 27651034

[B53] Salari E, Freudenburg ZV, Branco MP, Aarnoutse EJ, Vansteensel MJ, Ramsey NF (2019) Classification of articulator movements and movement direction from sensorimotor cortex activity. Sci Rep 9:14165. 10.1038/s41598-019-50834-5 31578420 PMC6775133

[B54] Seeber M, Michel CM (2021) Synchronous brain dynamics establish brief states of communality in distant neuronal populations. eNeuro 8:ENEURO.0005-21.2021. 10.1523/ENEURO.0005-21.2021 PMC8116110 33875454

[B55] Siegel M, Donner TH, Oostenveld R, Fries P, Engel AK (2008) Neuronal synchronization along the dorsal visual pathway reflects the focus of spatial visual attention. Neuron 60:709–719. 10.1016/j.neuron.2008.09.010 19038226

[B56] Sinai A, Crone NE, Wied HM, Franaszczuk PJ, Miglioretti D, Boatman-Reich D (2009) Intracranial mapping of auditory perception: event-related responses and electrocortical stimulation. Clin Neurophysiol 120:140–149. 10.1016/j.clinph.2008.10.152 19070540 PMC2819074

[B57] Tandon N (2012) Mapping of human language. In: Clinical brain mapping. (Yoshor D, Mizrahi E, eds), pp 203–218. New York: McGraw Hill Education.

[B58] Tandon N, Tong BA, Friedman ER, Johnson JA, Allmen GV, Thomas MS, Hope OA, Kalamangalam GP, Slater JD, Thompson SA (2019) Analysis of morbidity and outcomes associated with use of subdural grids vs stereoelectroencephalography in patients with intractable epilepsy. JAMA Neurol 76:672–681. 10.1001/jamaneurol.2019.0098 30830149 PMC6563575

[B59] Tong BA, Esquenazi Y, Johnson J, Zhu P, Tandon N (2020) The brain is not flat: conformal electrode arrays diminish complications of subdural electrode implantation, a series of 117 cases. World Neurosurg 144:e734–e742. 10.1016/j.wneu.2020.09.063 32949797

[B60] Torres D, Makarova J, Ortuño T, Benito N, Makarov VA, Herreras O (2019) Local and volume-conducted contributions to cortical field potentials. Cereb Cortex 29:5234–5254. 10.1093/cercor/bhz061 30941394

[B61] Trumpis M, Chiang C-H, Orsborn AL, Bent B, Li J, Rogers JA, Pesaran B, Cogan G, Viventi J (2021) Sufficient sampling for kriging prediction of cortical potential in rat, monkey, and human ECoG. J Neural Eng 18:e036011. 10.1088/1741-2552/abd460 PMC8058280 33326943

[B62] Vermaas M, Piastra MC, Oostendorp TF, Ramsey NF, Tiesinga PHE (2020) When to include ECoG electrode properties in volume conduction models. J Neural Eng 17:e056031. 10.1088/1741-2552/abb11d 33055363

[B63] von Ellenrieder N, Khoo HM, Dubeau F, Gotman J (2021) What do intracerebral electrodes measure? Clin Neurophysiol 132:1105–1115. 10.1016/j.clinph.2021.02.012 33773175

[B64] Womelsdorf T, Schoffelen J, Oostenveld R, Singer W, Desimone R, Engel AK, Fries P (2007) Modulation of neuronal interactions through neuronal synchronization. Science 316:1609–1612. 10.1126/science.1139597 17569862

[B65] Xing D, Yeh CI, Shapley RM (2009) Spatial spread of the local field potential and its laminar variation in visual cortex. J Neurosci 29:11540–11549. 10.1523/JNEUROSCI.2573-09.2009 19759301 PMC2910581

[B66] Young JJ, Friedman JS, Panov F, Camara D, Yoo JY, Fields MC, Marcuse LV, Jette N, Ghatan S (2019) Quantitative signal characteristics of electrocorticography and stereoelectroencephalography: the effect of contact depth. J Clin Neurophysiol 36:195–203. 10.1097/WNP.0000000000000577 30925509 PMC6493682

